# P-1059. The Impact of Low Influenza Immunization Rates on U.S. HealthCare Resources. A Modelling Assessment

**DOI:** 10.1093/ofid/ofaf695.1254

**Published:** 2026-01-11

**Authors:** Van Nguyen, Joaquin F Mould-Quevedo

**Affiliations:** VHN Consulting, Montreal, Quebec, Canada; CSL Seqirus Inc., Summit, NJ

## Abstract

**Background:**

Influenza significantly affects public health annually in the United States. Despite effective vaccines, consistently low immunization rates intensify influenza outbreaks, increasing outpatient visits and hospital admissions, ICU usage, and strain on healthcare infrastructure. This study aimed to quantify the impact of low influenza vaccination rates on U.S. health care resources.

**Methods:**

We applied a dynamic, age-stratified transmission model to estimate the effects of varying influenza vaccination rates during two representative U.S. influenza seasons: a low incidence (2011-2012) and high incidence (2017-2018). Four hypothetical immunization rates were evaluated (25%, 30%, 35% and 40%) considering outcome including outpatient visits, acute hospitalizations, number of hospital beds occupied and deaths. Vaccine effectiveness (VE) rate was averaged from CDC data over the last 10 seasons (42%). Vaccination rates by age group were estimated using CDC reports and this model assumed immunization with quadrivalent flu vaccines for all ages.

**Results:**

At the current U.S. flu immunization rate (≈35%) during a high flu incidence season the model project approximately 28,029,000 outpatient visits, 862 000 hospital admissions, 127,000 deaths with 221 000 acute hospital beds used and 34 000 ICU beds used. For a low influenza season, model projected 14,120,000 outpatient visits; 370,000 hospital admissions and 52,000 death deaths with 71 000 acute hospital beds used and 11 000 ICU beds used. Scenarios with an immunization rate below 45% consistently predicted substantial gain on hospital resources with saturation of ICU capacities in high-incidence seasons. Only increasing vaccination rate above 50% adequately mitigated healthcare resource saturation irrespective of seasonal influenza incidence.

**Conclusion:**

This modeling analysis highlights the urgent necessity to enhance U.S. influenza vaccination coverage. Aligning with the Healthy People 2030 initiative target of 70% vaccination coverage would significantly improve public health outcomes and prevent hospital resource saturation.

**Disclosures:**

Van Nguyen, PhD, CSL Seqirus: Advisor/Consultant|CSL Seqirus: Advisor/Consultant|CSL Seqirus Inc.: Advisor/Consultant Joaquin F. Mould-Quevedo, PhD, CSL Seqirus: Employee|CSL Seqirus: Employee|CSL Seqirus: Stocks/Bonds (Private Company)|CSL Seqirus: Stocks/Bonds (Private Company)|CSL Seqirus Inc.: Employee|CSL Seqirus Inc.: Stocks/Bonds (Private Company)

